# Zeolite-Polymer Composite Materials as Water Scavenger

**DOI:** 10.3390/molecules26164815

**Published:** 2021-08-09

**Authors:** Zakaria Tahraoui, Habiba Nouali, Claire Marichal, Patrice Forler, Julien Klein, T. Jean Daou

**Affiliations:** 1 IS2M (Institut de Science des Matériaux de Mulhouse, UMR 7361), Université de Haute Alsace (UHA), CNRS, 68100 Mulhouse, France; zakaria.tahraoui@uha.fr (Z.T.); habiba.nouali@uha.fr (H.N.); claire.marichal@uha.fr (C.M.); 2 Université de Strasbourg, 67081 Strasbourg, France; 3 APTAR CSP Technologies, 9 Rue de Sandholz, 67110 Niederbronn-les Bains, France; patrice.forler@aptar.com (P.F.); julien.klein@aptar.com (J.K.)

**Keywords:** zeolite, cationic exchange, exchanged zeolite-based composite, water adsorption, humidity scavenger

## Abstract

The influence of the charge compensating cation nature (Na^+^, Mg^2+^) on the water adsorption properties of LTA-type zeolites used as filler in composite materials (zeolite/polymers) was investigated. Large scale cation exchanges were performed on zeolite powder at 80 °C for 2 h using 1 M magnesium chloride (MgCl_2_) aqueous solutions. XRF, ICP, and EDX analyses indicate a successful cationic exchange process without the modification of the zeolite structure as shown by XRD and solid-state NMR analyses. Composite materials (granulates and molded parts) were manufactured using to extrusion and injection processes. In the case of MgA zeolite, nitrogen adsorption–desorption experiments allowed us to measure a microporous volume, unlike NaA zeolite, which is non-porous to nitrogen probe molecule. SEM and EDX analyses highlighted the homogeneous distribution of zeolite crystals into the polymer matrix. Water adsorption capacities confirmed that the trends observed in the zeolite powder samples are preserved after dragging zeolites into composite formulations. Granulates and molded parts composite samples containing the magnesium exchanged zeolite showed an increase of their water adsorption capacity up to +27% in comparison to composite samples containing the non-exchanged zeolite. The MgA composite is more promising for water decontamination applications due to its higher water adsorption properties than the NaA composite.

## 1. Introduction

Water adsorption by porous solids is important for many applications that involve the capture and release of water, such as electric dehumidifier, adsorption heat pumps (AHPs), alcohol/organic solvent dehydration, etc. One of the most promising AHPs technologies in this context is based on the evaporation and consecutive adsorption of water under specific conditions. The first prototypes of adsorption heat pumps/cooling used an adsorbent made of zeolite loose grains [1,2]. Water content in natural gas is also considered as a critical concern because it can cause corrosion and hydrate formation, ending in pipeline blockage [3,4].

Moreover, to improve the quality and safe storage of processed foods and moisture sensitive materials, the need for moisture removal technology is also becoming important [5,6,7]. In daily human life, relative humidity is also an important factor, as it affects health. A highly humid environment favors house dust mites, provides a favorable environment for fungi and harmful bacteria to grow, destroys the heat–humidity balance of human body, etc. [8]. Therefore, the demand to control humidity through the development of highly efficient sorbent technology has enhanced the interest in new porous materials, especially microporous materials [9].

A variety of porous materials (zeolites, metal organic frameworks, clays, carbon based adsorbents, organic polymers) have been explored for all of these applications, but it still remains a challenge to find low-cost and high-performance materials combining high water uptake, precise operational pressure range control, recyclability, stability, etc. [10,11,12,13]. Zeolites are widely used for molecular decontamination due to their high adsorption properties and their thermal, chemical, and mechanical stabilities [14,15,16,17,18]. Zeolites are crystalline aluminosilicates with a 3-dimensional, open anion framework consisting of oxygen-sharing SiO_4_ and AlO_4_^−^ tetrahedra [19,20,21]. Each silicon ion has its +4 charge balanced by four tetrahedral oxygens, and the silica tetrahedra are therefore electrically neutral. Each alumina tetrahedron has a residual charge of −1 since the trivalent aluminum is bonded to four oxygen anions. Therefore, each alumina tetrahedron requires a +1 charge from an extraframework cation in the structure to maintain electrical neutrality [20]. These cations are usually sodium ions that are present in the synthesis medium [22]. Sodium ions can be easily exchanged by mono or divalent cations. Several studies have mentioned the major role that these cations (Na^+^, K^+^, Li^+^, Mg^2+^, Ca^2+^, Zn^2+^, Mn^2+^) can play in increasing the affinity between the adsorbates and the adsorbents or in modifying the separation properties of the zeolites towards gases [12,19,23,24,25].

In addition, it is well known that aluminosilicate zeolites containing compensating cations in their framework show a highly hydrophilic character (especially the one with low Si/Al ratio), which gives them a strong affinity towards water molecules. The most commonly employed zeolites for water adsorption in industry are the 3A (KA) and 4A (NaA) zeolites (LTA-type zeolite) and the 13X (NaX) zeolite (FAU-type zeolite) [26,27,28,29,30,31,32,33].

The aluminosilicate framework of zeolite A (LTA-type) can be described in terms of two types of polyhedral: one being a simple cubic arrangement of 8 polyhedra (the double 4-rings), and the other being a truncated octahedron of 24 tetrahedra also called the β- cage. In LTA, sodalite cages are joined via double 4-rings, creating an α-cage in the center of the unit cell. Alternatively, the framework can be described as a primitive cubic arrangement of α- cages joined through single 8 member rings (MR) [34]. Zeolite A has a three-dimensional pore system, and molecules can diffuse in all three directions in space by moving across the 8 MR windows that are about 0.42 nm diameter and that connect the cavities. The size of the pore openings depends on the size of the charge compensating cations. Normally, zeolite A is synthesized in the Na-form, which has a pore opening of about 0.4 nm. The sodium cations can then be exchanged, thereby tuning the size of the pore openings.

All reported works agree that water adsorption in zeolites is mainly directed by interactions between water, charge compensating cations, and the zeolite framework. Depending on the charge (mono or divalent cations) and the kinetic diameter of the compensating cation, the available microporous volume and the accessibility to the micropores are modified, which leads to different adsorption behaviors and adsorption capacities [28]. Although LTA-type zeolites show attractive adsorption uptake and high water affinity, their global performance regarding water adsorption is still not optimal, mostly due to their nature, size, and affinity to the water molecules of the compensating cations. Therefore, there is still a considerable need to improve zeolite adsorbents for this targeted application.

Depending on the application, the powder form of zeolites can be problematic, especially for applications involving adsorption [35,36,37,38,39]. Indeed, interest is increasingly focused on composite materials based on zeolites as filler, which can preserve the zeolite performance and limit pollution problems related to powder spreading [40].

The polymeric membrane for gas separation such as natural gas sweetening, landfill gas recovery, hydrogen recovery and purification, flue gas, and air separation is one of the most known applications that uses zeolite materials dispersed into a polymer matrix. Used as a filler, zeolites confer a certain permeability and selectivity to the composite material [41] due to their shape discrimination resulting from their narrow pore distribution [41,42,43]. As for the powder form, zeolite composite can be used for the removal of polluting cations present in wastewater [44,45]. Patents reporting drying solutions based on zeolitic composite materials are mentioned in the literature [46,47], all of which are trying to exploit the zeolite performance once it is blended into polymers.

In this work, NaA (LTA-type) zeolite provided by APTAR CSP Technologies was exchanged using a MgCl2 aqueous solution. The cation choice was made to have the smallest divalent cations in order to maximize the available microporous volume. The parent zeolite (NaA) and the exchanged zeolite (MgA) samples were then used as fillers to produce composite (zeolite/polymers) materials.

The prepared samples (zeolite powders and composite materials) were then fully characterized by XRF, ICP, SEM, and XRD, and their adsorption performances were systematically evaluated by comparing their nitrogen and water adsorption capacities to the parent zeolite powder (NaA).

## 2. Results and Discussion

### 2.1. Chemical Composition

The chemical composition of the raw and exchanged magnesium zeolite sample was determined through X-ray Fluorescence analysis, and the results are reported in Table 1.

The LTA zeolite framework consists of strictly alternating silicon and aluminum atoms, leading to a Si/Al ratio of 1, the minimum allowed by Lowenstein’s rule [48], which forbids two aluminum tetrahedra to be linked. The chemical analysis in Table 1 shows that the Si/Al ratio obtained is around 1 for the raw LTA-type zeolite, which is in agreement with the expected value from the literature [49,50]. It is worth noting that the cations/Al ratio in the raw LTA-type zeolite as well as for the MgA sample is slightly greater than 1 (NaA = 1.09 and MgA = 1.05), probably due to a slight excess of aggregated cations on the surface of the zeolite compensating the negative charge due to the presence of a small amount of defects (see NMR section) or due to the presence of Mg(OH)_2_ particles, as already observed in the literature [51,52,53].

**Figure 1:**
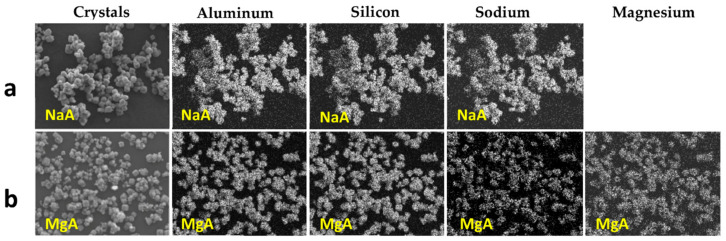
EDX mapping of aluminum, silicon, and sodium in (**a**) NaA and of aluminum, silicon, sodium, and magnesium in (**b**) MgA powder samples.

**Figure A1:**
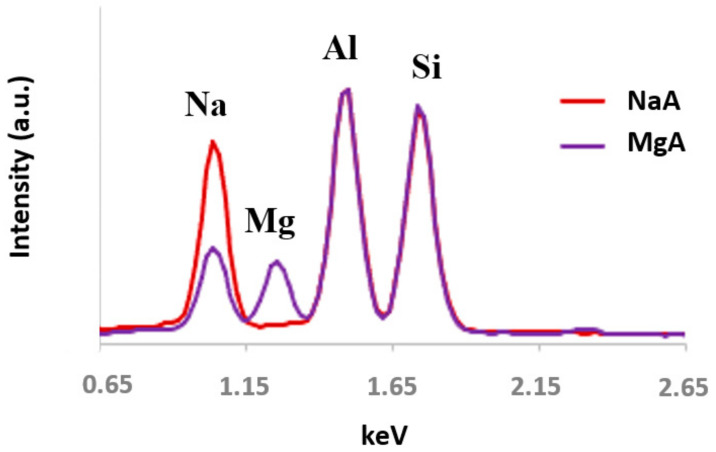
EDX spectra of NaA and MgA samples.

The concomitant sodium loss and magnesium increase (as seen in EDX mapping and analysis, see Figure 1 and Figure A1, respectively) is an indication of the successful exchange process. For the MgA sample, 55% of the compensating positive charges (sum of present cations) seem to be due to the magnesium atoms (see Table 1).

However, some studies have mentioned a complete cationic exchange [28,54]. The fact that no complete cationic exchange is observed in this work could be attributed to the lack of exchange steps and/or to the difficulty of exchanging the sodium cations present in the sodalite cages. Indeed, depending on the cation size and its hydration sphere, the sodium cations located in the sodalite cages (site I) are difficult to extract because of the small cage aperture (6 MR), which has an opening of 2.2 Å [55,56]. Nevertheless, in the case of water adsorption, the main goal of our study, a 100% Na exchange is useless since water is not adsorbed in sodalite cages, as the kinetic diameter of water molecule (2.65 Å) [57] is larger than the pore opening of sodalite cages [55].

It is worth noting that according to XRF analysis, this cationic exchange process occurs without modifying the global Si/Al molar ratio of both zeolites, making it an efficient technique for the chemical modification of zeolites.

The elemental distribution of Si, Al, Na, and Mg in the samples was studied using EDX mapping, which is displayed in Figure 1.

According to Figure 1, each white pixel shows the presence of the corresponding atom. The loss of sodium cations in favor of the exchanged cation (Mg^2+^) is confirmed by the loss of intensity (whiteness) between the EDX Na mapping shown on the exchanged materials compared to the one of the raw materials. Figure 1 also shows a uniform distribution of Mg^2+^ cations in the crystal particles, which indicates the success of the exchange process.

In agreement with both of the XRF analyses (see Table 1), the EDX analysis displayed in Figure A1 also reports a loss of sodium cations after exchange in favor of the selected new cation, Mg^2+^, which is an additional indication of successful cationic exchange.

### 2.2. Chemical Structure

**Figure 2:**
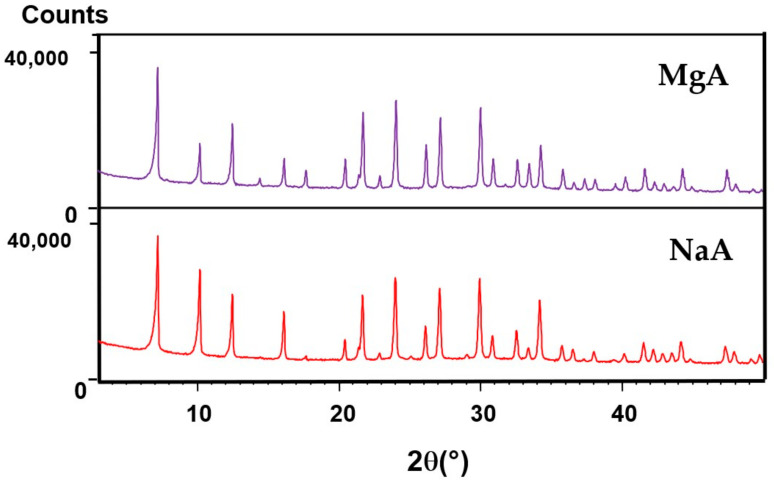
XRD patterns of the raw LTA-type zeolite and its associated MgA form.

The XRD patterns of the raw and exchanged LTA-type zeolites are displayed in Figure 2. All samples showed a single LTA-type zeolite phase, which is in agreement with the corresponding patterns available in the literature [49] (Pattern 04-016-9920 from ICDD). The unit cell parameters (a, b, and c) of the LTA-type zeolite with a cubic system and Fm-3c as space group were determined with the X’Pert HighScore and STOE Win XPOW software programs [58] according to Werner algorithm [59]. For the raw LTA material, a = b = c are equal to 24.57 Å, which is in agreement with the literature [60]. After cationic exchange, the unit cell parameters decrease (a = b = c = 24.53 Å). The smaller cation radius of magnesium (0.72 Å) in comparison with sodium (1.02 Å) [61], leads to a structure contraction as well as slight changes in peak intensity ratios and shifts when the sodium cations are replaced by other alkali or alkali earth metal cations. These results are in agreement with the literature [62,63,64] and are explained as a consequence of the difference between the scattering power, which is specific to each cation, and the slightly different occupation sites in the pores [64,65].

This is also an indication of a successful cation exchange. Despite those observations, all of the XRD patterns of the exchanged samples are similar to those of the parent materials, indicating that cationic exchange did not significantly affect the structure at a long-range scale.

### 2.3. Crystals Morphology

The crystallized phase with the cubic morphology characteristics of LTA-type zeolites and a particle average size around 3 μm are observed. After cation exchange with the Mg^2+^ cations, the morphology of the crystals is preserved, but small particles pointing towards a potential Mg(OH)_2_ species seem to be randomly present on the surface of some crystals in negligible amounts.

### 2.4. Local Chemical Structure

**Figure 3:**
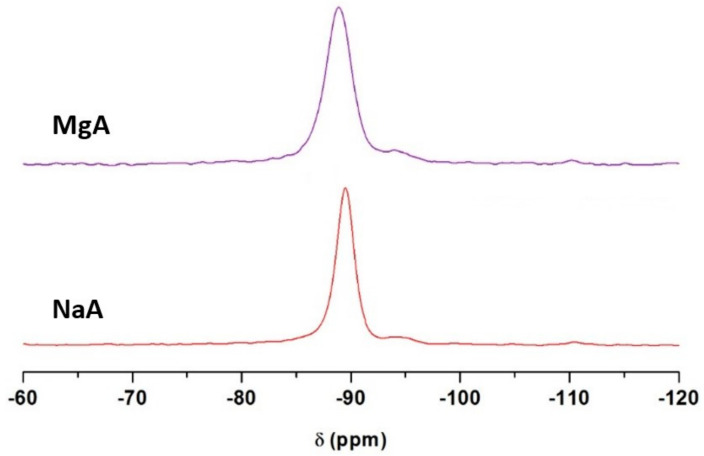
^29^Si MAS NMR spectra of NaA and MgA samples. Only the isotropic region is shown.

^29^Si and ^27^Al MAS NMR were performed to study the local environments of the corresponding atoms before and after cationic exchange. ^29^Si MAS NMR spectra of NaA and MgA samples are displayed in Figure 3.

One main resonance is detected at −89.5 ppm and −88.9 ppm for the NaA and MgA samples, respectively, corresponding to tetrahedral Si(OAl)_4_ species typical of the LTA-type zeolite [66].

An additional resonance is seen around −94.4 ppm and −94.2 ppm for the NaA and MgA samples, respectively, accounting for 5.1% and 3.9 % of the total signal [67]. This small peak of similar amounts, regardless of the sample (error of 5%), could be attributed to a Si(OSi)(OAl)_3_ species that is already present in the parent material sample (NaA) [68]. A third resonance is observed at around −110.5 ppm and −110.2 ppm for the two samples, accounting for approximately 1% of the total signal. This small peak can correspond to amorphous Si(OSi)_4_ species that are still present after the exchanges have taken place.

A broadening of the main signal is observed after cationic exchange. The width at half height increases from 130 Hz for the NaA raw sample to 170 Hz for the MgA sample. This could result from a change in the cation distribution of the environments around the Si atoms. Indeed, since this is a not a total exchange, both magnesium and sodium cations coexist within the framework. Magnesium cations are divalent; thus, the required amount and the location of the cations in the structure change in comparison to sodium cations, which leads to several potential environments for silicon atoms. The observed broadening could also result from heteronuclear dipolar interaction between the silicon atoms and the Mg^2+^ charge compensating the cations if both are close [67].

In parallel, the position of the main resonance is shifted from −89.5 ppm to −88.9 ppm after the exchange with magnesium. This shift is probably related to the contraction of the unit cell, as mentioned in the XRD section. This phenomenon was already observed by Price et al. [64] since the ^29^Si chemical shift is known to depend on the bond lengths and bond angles.

**Figure 4:**
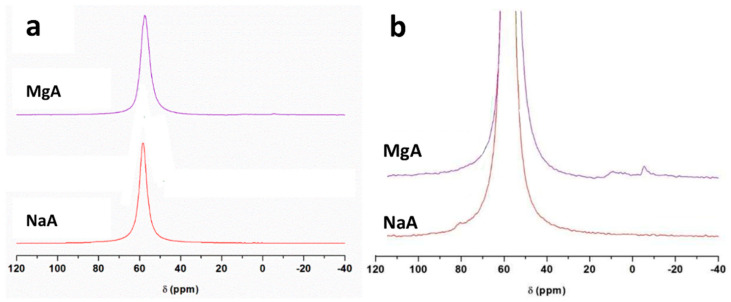
^27^Al MAS NMR spectra of (**a**) NaA and MgA powder samples and (**b**) a zoom of (**a**). Only the isotropic region is shown.

Figure 4a,b displays ^27^Al MAS NMR spectra of the NaA and MgA samples.

One main resonance is detected at 58 ppm for the NaA samples and at 55 ppm for the MgA samples corresponding to tetrahedrally coordinated aluminum Al(OSi)_4_, as expected for the LTA-type zeolite [66]. After exchange, a broadening of the main resonance is observed for the MgA sample (535 Hz in comparison to 450 Hz for the NaA sample), indicating a distribution of the environment around the Al atoms. The ^27^Al MAS NMR spectra of the magnesium exchanged sample also present weak signals from 6 ppm to −5 ppm and at around −15 ppm, accounting for approximately 2% of the total signal that could correspond to pentahedral and octahedral aluminum atoms, respectively. A small extraction of aluminum atoms from the zeolite framework seems to occur during the exchange step.

^29^Si and ^27^Al MAS NMR spectra are sensitive to the cationic exchange of Na^+^ by Mg^2+^, indicating slight modifications of both the silicon and aluminum environments. However, the resonance characteristics of LTA-type zeolites are observed to be in agreement with the XRF, XRD, and SEM conclusions.

### 2.5. Textural Properties

**Figure 5:**
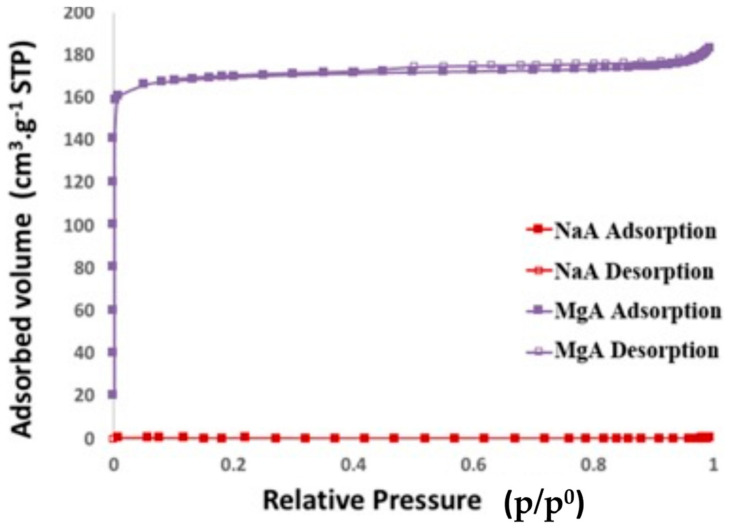
Nitrogen adsorption–desorption isotherms of the raw LTA-type zeolite and its associated magnesium form at −196 °C.

The nitrogen adsorption–desorption isotherms of the raw and exchanged LTA-type zeolites with Mg^2+^ are displayed in Figure 5.

The textural properties (BET surface and microporous volume) of all of these samples are shown in Table 2.

As expected, no N_2_ adsorption is observed for the NaA sample. This phenomenon was already mentioned in the literature [54,69,70,71] and is due to the position of the Na^+^ cations near the pore opening, which obstruct the accessibility of N_2_ to the microporosity.

In contrast, the magnesium exchanged sample (see Figure 5) allows nitrogen diffusion through the porosity of the LTA-type zeolite and exhibits a type I isotherm according to the IUPAC classification of isotherms [72]. Since the samples were in powder form, the adsorbed volume that is observed between p/p^0^ = 0.9 and 1 is attributed to the inter-particular porosity.

When the parent LTA-type zeolite is exchanged with magnesium, a capacity up to 170 cm^3^·g^−1^ S.T.P. (Standard Pressure and Temperature) of nitrogen is observed, representing a microporous volume of 0.26 cm^3^·g^−1^ and a surface area of 693 m^2^·g^−1^. This could be attributed to a less congested accessible microporous volume due to the cation positions, as already observed in our previous paper [26].

### 2.6. Water Adsorption Properties

**Figure 6:**
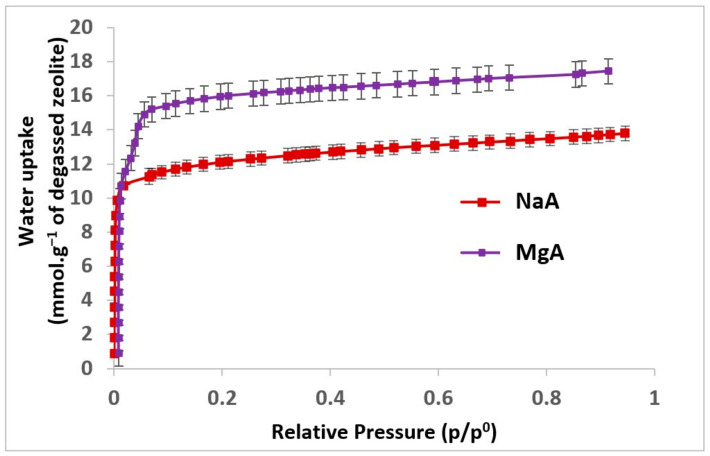
Water adsorption isotherms of the raw LTA-type zeolite and its associated magnesium form at 25 °C.

The water adsorption isotherms of the raw and exchanged LTA-type zeolites are displayed in Figure 6. The water adsorption capacities were determined to be at p/p^0^ = 0.2 (representing the adsorption in the microporosity of the samples) and are reported for each sample in Table 2.

According to Figure 6, water adsorption is observed for each zeolite sample regardless of the nature of the compensating cation. In the case of nitrogen molecules, no adsorption is observed for the sodium forms since the cation obstructs the pore opening. However for water molecules, which have a smaller kinetic diameter than that of nitrogen (2.65 Å in comparison to 3.64–3.80 Å) [57], diffusion through pore openings is possible. The water adsorption capacity for the NaA sample (21.8 Wt.%) displayed in Table 2 is in agreement with the literature [32,73]. A gain in the water adsorption capacity is observed for the MgA sample (28.8 Wt.%) in comparison to the NaA sample.

The results displayed in Table 2 represent for MgA sample show an increase in the water uptake of 32% in comparison to the associated raw sample. This increase can be explained by the size difference of the cation (Mg^2+^: 0.72 Å, Na^+^: 1.02 Å) and by the divalent nature of the magnesium cation. With this cation, only a half of the replaced monovalent cations are needed. Thus, both the size difference and the number of required cations contribute to the gain of accessible microporous volume, explaining the significant increase of water uptake for the MgA sample. However, the interactions with the compensating cations (solvating layer) also have to be taken into account. Here, bivalent magnesium cations will have higher affinity with water molecules due to their two positive charges and thus will also have a higher sphere of hydration than monovalent sodium cations [61,74]. In their work, Crupi et al. mentioned the reduction of steric hindrance offered by the Mg-form of LTA due to the magnesium cations located at the edge of the α-cage leaving free porosity. This cation distribution seems to thus favour the formation of a strong tetrahedral network of water molecules, which could reduce the diffusion of the molecules that are involved in these interactions [75,76].

The higher available microporous volume in addition to a higher hydration sphere leads to an increase in water uptake.

These results clearly indicate that increasing the available microporous volume facilitates the storage of water molecules and that using magnesium as a compensating cation shows higher water uptake performance [26].

In the second part of this work, these exchanged zeolites are used as a filler to develop composite materials. The results of this work are displayed in the next section.

### 2.7. Composite Materials

**Figure A2:**
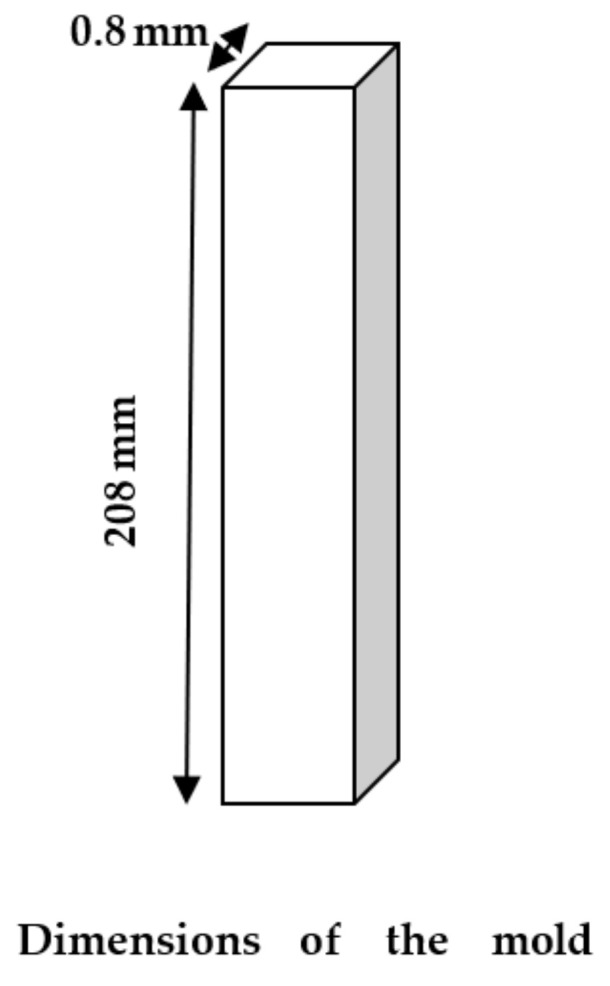
Mold cavity dimensions.

Composite materials in molded part forms were obtained from the granulates by filling the mold of the injection equipment displayed in Figure A2. The detailed dimensions of the obtained composite material samples are shown in Table 3.

All of the formulations showed similar behaviors during the shaping processes, both for extrusion and injection. From a macroscopic point of view, the dimensions of the molded parts containing zeolite as filler was shorter than those of pure polypropylene (210 mm length). Indeed, the presence of zeolite modified the fluidity of the melt material, which could not completely fill the mold cavity. Moreover, for all of molded parts with zeolite-based formulations, no significant differences were observed in terms of size, color, or texture.

After being manufactured, each sample was transferred into sealed aluminum bags in order to avoid potential environmental pollution.

All of the obtained composite materials were then fully characterized. The next section reports the characterization results and compares them with the properties of the parent zeolites.

#### 2.7.1. Determination of Zeolite Content in Each Composite Materials

Since the adsorption properties of the composite materials are related to zeolite contents, zeolite loading is one of the most crucial points to be controlled. To determine the zeolite loading, a calcination was conducted as described in the characterization technique section. By weight difference after a calcination, the zeolite loading of the granulates and the weight of the molded parts were determined.

According to the results obtained from the granulates (ranging around 60 Wt.%), the maximal deviation from the target loading was 3.2 Wt.%. This deviation does not seem surprising since the mixing and blending processes are not optimized for the blending of heterogeneous materials such as granulates (polymers) with zeolite powders. In these processing conditions, the determined zeolite contents of the composite materials seemed globally consistent with the amounts of zeolites introduced into the formulations during their preparation.

After the injection process, which used composite granulates as feed material, zeolite loadings around 60 Wt.% seemed more consistent with the target loading.

From a macroscopic point of view, the composite materials seemed homogeneous in terms of the dispersion of the zeolite in the polymer matrix. However, in order to garner some insight into the sample homogeneity from a microscopic point of view, Scanning Electron Microscopy (SEM) was performed on each sample.

#### 2.7.2. Composite Morphology and Homogeneity

**Figure 7:**
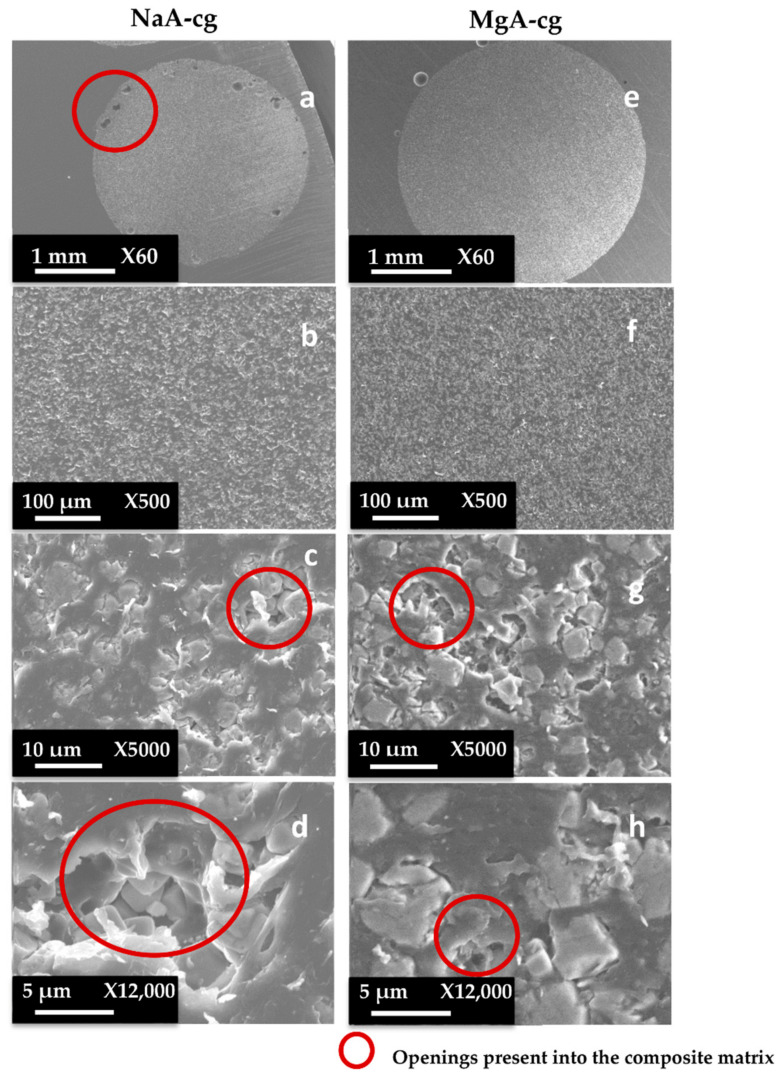
SEM images at different magnifications of (**a**–**d**) NaA-0-cg and (**e**–**h**) MgA-1-cg samples.

SEM images of composite materials in the form of granulates (NaA-cg and MgA-cg samples) are displayed in Figure 7.

Figure 7 shows a homogeneous distribution of zeolite crystals within the polymer matrix at low and high magnification. In addition, the different samples (NaA-cg and MgA-cg) seem homogeneous with each other in terms of zeolite distribution.

Figure 7a shows the presence of a macroporosity composed of holes in the order of 10 µm to several tens of micrometers for the NaA-cg sample, while no holes of this size are observed for the MgA-cg sample (see Figure 7e). The presence of air during the extrusion process could explain the size and the shape of these observed holes, which are internally similar to the bulk of the granulates lined with polymers and zeolites.

Figure 7c shows the presence of small holes in each material, ranging from the size of the zeolite crystals (below 5 µm) to slightly higher ones (up to 10 µm for agglomerated crystals). Some of these openings can be caused by the polishing step, whereas others seem to be specific to the global composite material. In the end, this observation seems homogeneous for all samples. These holes in the composite material samples that are also observable in Figure 7g could be the path for the water molecules that can be adsorbed by the distinguishable zeolite crystals. The cubic morphology of the zeolite crystals could be slightly affected by the polishing step, which could break crystals. Figure 7d allows us to see the preserved cubic morphology of some of the crystals observed within the porosity.

The same sample preparation method was used in order to observe the slice (side view) of the molded parts.

**Figure A3:**
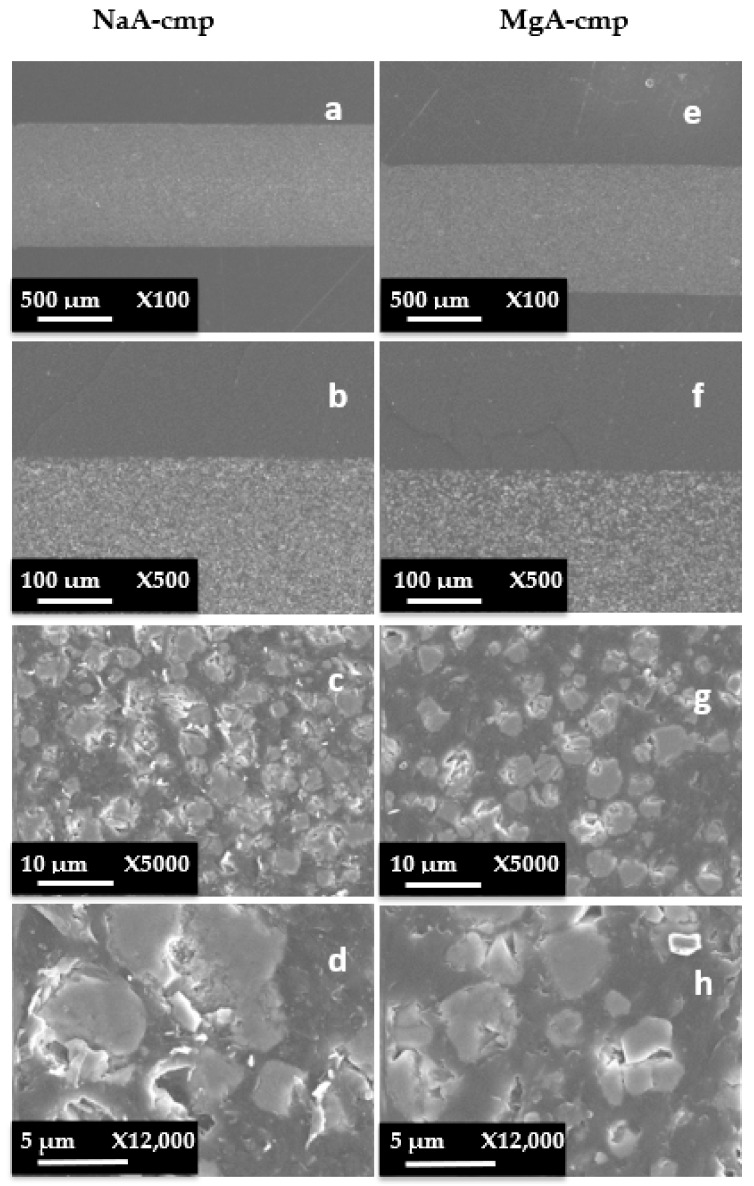
SEM images at different magnifications of molded part forms (side view) of (**a**–**d**) NaA-cmp and (**e**–**h**) MgA-cmp samples.

SEM images of composite materials in form the of molded parts (side view) (NaA-cmp and MgA-cmp samples) are displayed in Figure A3.

Figure A3a–d shows a homogeneous distribution of the zeolite crystals within the polymer matrix at low and high magnification for all samples. The observation of the side view confirms that homogeneity is also conserved within the composite and not only at the surface.

Figure A3c,d,g,h also shows the presence of small holes, ranging from the size of the zeolite crystals (2 to 5 µm) to higher ones for all samples. The manufacturing of molded part composite materials seems to preserve these holes, which could be the path for the water molecules to be adsorbed in zeolites, as already mentioned. Finally, the front view of the molded parts was investigated. There was no previous preparation. The samples were observed as they were obtained without the further use of a resin and polishing step.

**Figure A4:**
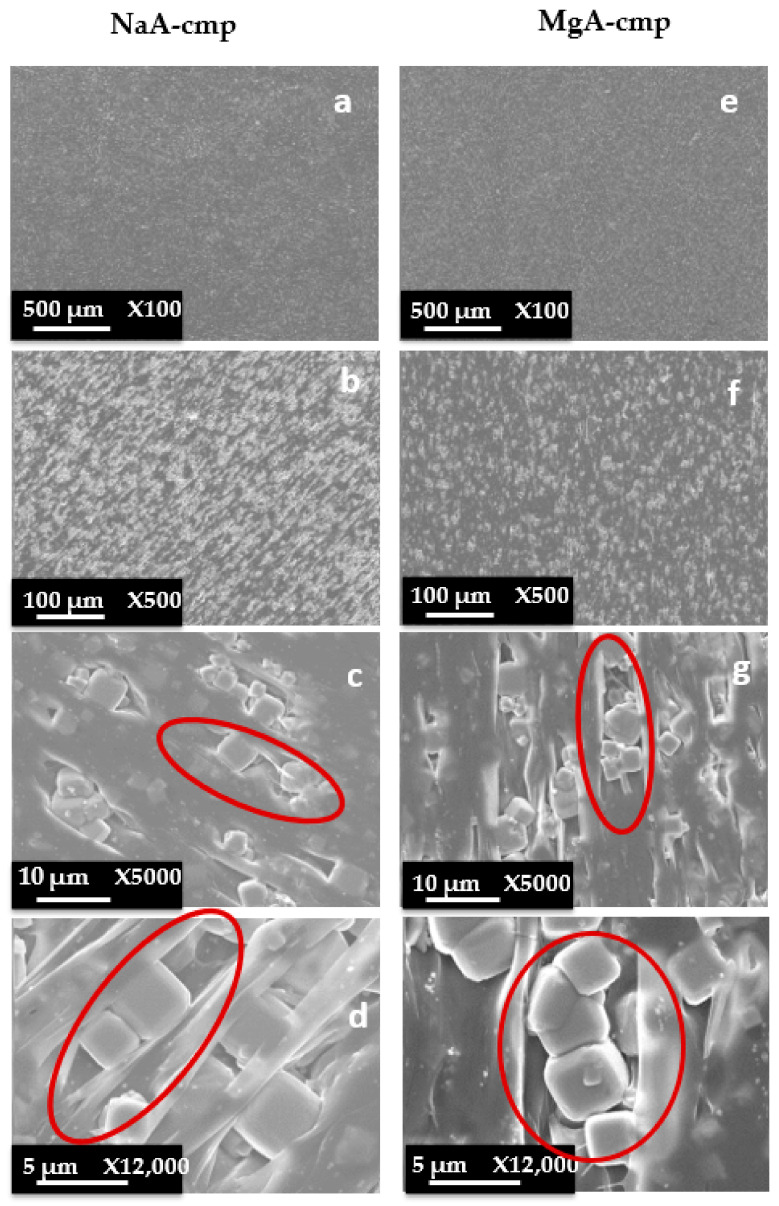
SEM images at different magnifications of molded part forms (top view) of (**a**–**d**) NaA-cmp and (**e**–**h**) MgA-cmp samples.

SEM images of composite materials in the form of molded parts (top view) (NaA-cmp and MgA-cmp samples) are displayed in Figure A4. Figure A4a–d shows a homogeneous distribution of zeolite crystals within the polymer matrix at low magnification.

By increasing the magnification, Figure A4c,d,g,h shows the zeolite crystals inside the porosity of the composite materials for the NaA-cmp and MgA-cmp samples. The cubic morphology of the zeolite crystals is distinguishable. A preferential orientation of the polymer fibers is clearly seen at high magnification regardless of the zeolite. These observations can be explained by the injection process, which pushes the formulation in its molten state into the mold cavity. The trajectory of the fluid material seems to influence the orientation of the polymer fibers and the distribution of zeolite crystals. Indeed, since there was no sample modification prior to the observation, those pictures show the material as it was prepared.

**Figure A5:**
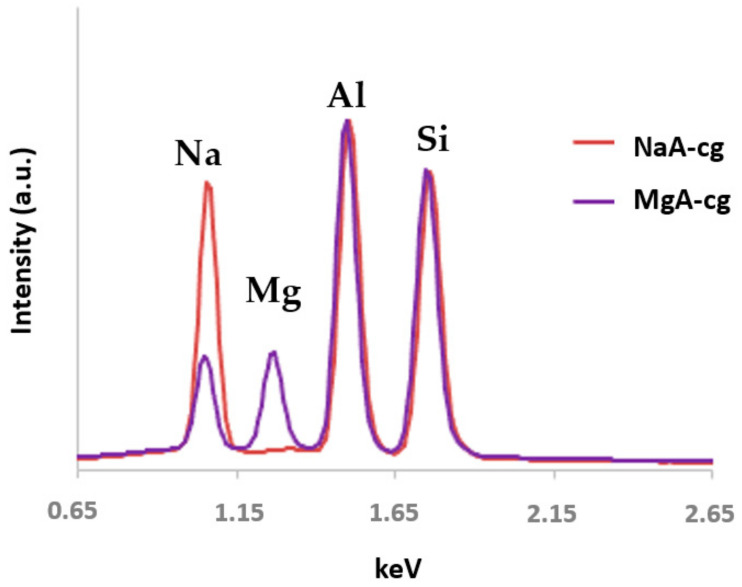
EDX spectra of NaA-cg and MgA-cg samples.

**Figure A6:**
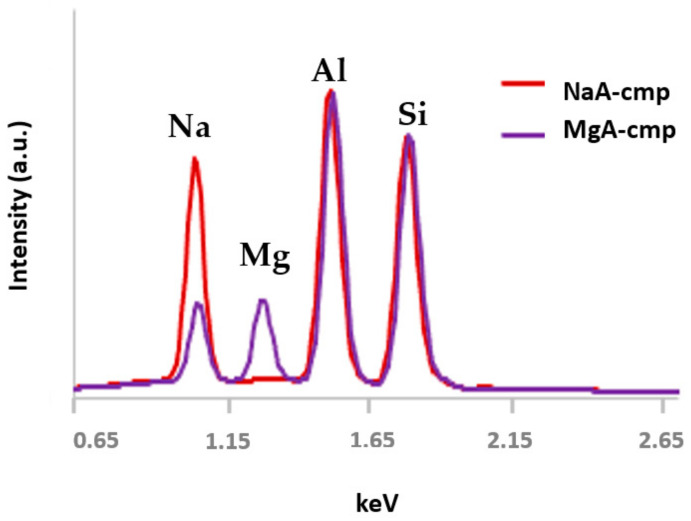
EDX spectra of NaA-cmp and MgA-cmp (side view) samples.

The elemental distribution of Si, Al, Na, and Mg in the composite samples (granulates and side view of the moulded parts) was studied using EDX mapping. The results are displayed in Table 4 and Table 5. EDX analysis results are displayed in Figure A5 and Figure A6.

According to Figure A5 and Figure A6 in comparison to Figure A1, the elemental composition of the different samples, NaA and MgA (powders), seems to be maintained after being mixed with polymer components and after being manufactured in the granulate and molded part forms using the processes described above.

Table 4 and Table 5 represent the elemental mapping of the different samples and clearly distinguish the polymer components composed of carbon from the inorganic parts representing the zeolite crystals composed of silicon, aluminum, etc. In addition, the localization of the different atoms confirms the homogeneous distribution as well as the success of the exchange process with the magnesium cations.

The observation of the different samples by SEM and EDX analysis showed the homogeneous repartition of the zeolitic particles into the polymer matrix.

#### 2.7.3. Water Adsorption Properties of the Elaborated Composite Materials

**Figure 8:**
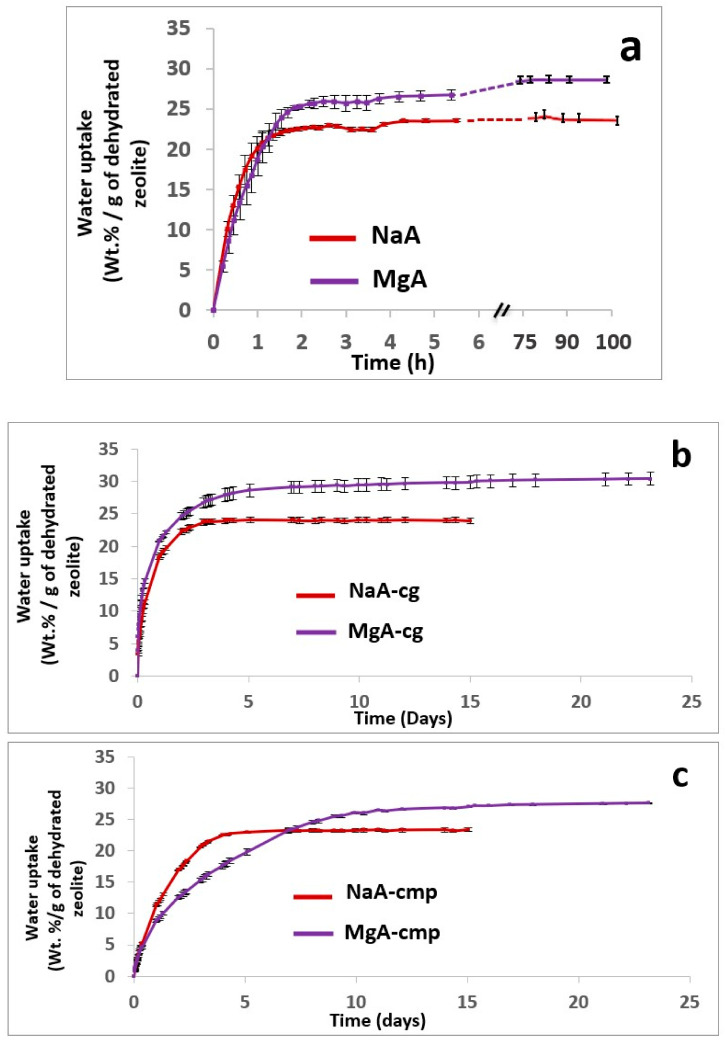
Water adsorption kinetics at 25 °C and Patm of (**a**) the LTA-type zeolite (NaA) and (MgA) and their associated composites forms, (**b**) NaA-cg and MgA-cg samples, and (**c**) NaA-cmp and MgA-cmp samples.

Prior to testing the water adsorption properties of the composite material samples, the associated zeolite powder samples were analyzed in the same conditions as those from the referenced studies. The adsorption kinetic curves of the zeolites and their corresponding composites are displayed in Figure 8. The adsorption capacities are compiled in Table 6.

As expected from the water adsorption isotherms of the zeolite powder displayed in Figure 8, all samples showed water adsorption capacity, but some differences were observed in the time needed to reach saturation. The saturation plateau was reached after about 2 h for the NaA and MgA zeolite powder samples, while for composite materials, regardless of their shape, it took at least 5 days to reach the plateau (Figure 8). In all cases, the adsorption kinetic was faster in the powder forms in comparison to its associated composite samples. The molded samples presented lower kinetic of water adsorption than the granulates. The diffusion of the water molecules inside the molded composite and therefore the polymers and zeolite seemed to be slower, as shown by the slope of the kinetic curve (Figure 8c).

According to Table 6 and Figure 8, all of the composite materials show a water adsorption capacity similar to the parent zeolites NaA and MgA (when reported to the dehydrated zeolite content), meaning that the water adsorption capacity of the zeolite is not affected by the polymer matrix. The similarity of the water adsorption capacities between the zeolite powders and the corresponding composite materials suggests that water is most likely adsorbed into the zeolite crystals.

Globally, the MgA zeolite in both its powder and composite forms (MgA-cg and MgA-cmp) shows a significant increase in water adsorption capacity in comparison to the sodium form by up to + 22%, 27%, and 18% for the powder, granulate, and molded part forms, respectively.

As already mentioned earlier in this work, the cationic exchange enhances the water adsorption capacity of the zeolites, which seems here to be preserved after dragging the zeolites into polymeric formulations.

## 3. Material and Methods

### 3.1. Materials

#### 3.1.1. Zeolites

LTA-type zeolite (NaA) was provided in its powder form by APTAR CSP Technologies (Niederbronn Les Bains, France). Magnesium chloride (MgCl_2_.6H_2_O, ACS-ISO for analysis >99%) salt was purchased from Carlo Erba and was used for the ion exchange process.

#### 3.1.2. Polymers

The polymer formulation provided by APTAR CSP Technologies (confidential) was based on the use of a polymer mixture with polypropylene as a main component [46]. The polymer formulation was mixed with 60 Wt.% of zeolite (raw or exchanged) powder.

### 3.2. Large Scale Cation Exchange

NaA zeolite was modified by exchanging a part of the sodium compensating cations present in the parent zeolite framework with magnesium (Mg^2+^) cations through a cationic exchange process using 1 M MgCl_2_ aqueous solution.

**Figure A7:**
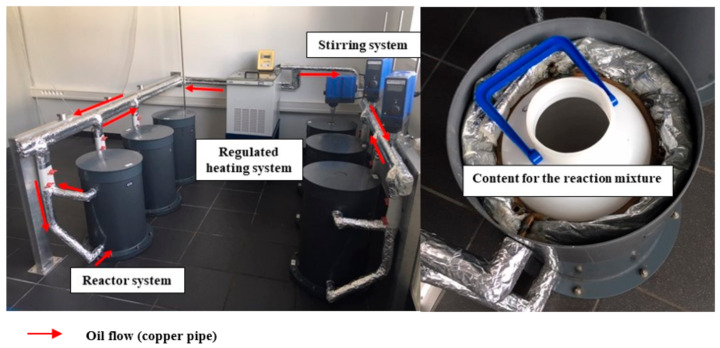
Large scale synthesis equipment.

A total of 10 h before the experiment, the containers for the reaction mixtures (polypropylene bottles of 10 L) containing 7.5 L of demineralized water were placed into the dedicated equipment (See Figure A7 in Appendix A) and were heated to let the temperature stabilize at 80 °C. The day of the experiment, 1 M MgCl_2_ aqueous solution was prepared by adding MgCl_2_ salt (1524.83 g) to the heated 7.5 L of demineralized water through stirring. The pH value of this electrolyte aqueous solution was found to be around 5.5.

The raw hydrated zeolite (more or less exactly 468.75 g) was then blended with the 1 M electrolyte aqueous solution. The mass ratio of the reaction mixture was 1 g of dehydrated zeolite for 20 mL of electrolyte aqueous solution. The pH value of this mixture was around 8.5.

The reaction mixture was then maintained at 80 °C for 2 h by stirring. Depending on the amount of zeolite required, several bottles could be used simultaneously.

After 2 h, the reaction mixtures (30 L = 4 bottles per sample) were washed all at once with cold demineralized water (60 L) in a 100 L polypropylene container under mechanical stirring (10 min). The mixture was then filtered using a Rousselet-Robatel RC40VXR centrifuge (at 3000 rpm until no more wastewater was observed, 5–10 min). A total of 60 L of additional demineralized water was sprayed onto the agglomerated zeolite for more washing. Centrifugation was stopped after no more wastewater was observed, indicating that the zeolite powder was globally dry. All of the samples were then dried at 100 °C for 3 days.

The obtained exchanged zeolite sample was denoted as follows: cA with c, the major compensating cation. The sample was fully characterized.

### 3.3. Activation of Zeolite Powders Used for Composite Manufacturing

**Figure A8:**
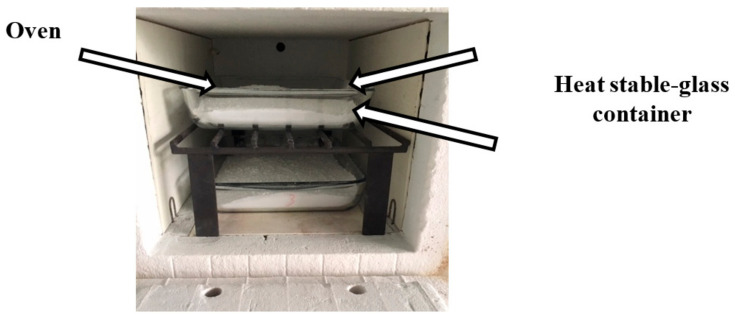
Dehydration of zeolite powder materials in the oven.

Prior to being used for composite manufacturing, zeolite powders were dehydrated in order to activate their adsorption properties. To do that, zeolite powders were placed in a Pyrex glass tray (1.8 Kg of zeolite per glass tray, see Figure A8 in Appendix A), and the temperature program displayed in Table 7 was applied using a Nabertherm Controller B170 oven. After being dehydrated, the zeolite powders were transferred into moisture tight aluminum bags to preserve their adsorption properties, and the bags were then sealed.

### 3.4. Elaboration of Composites

Before being shaped into composite forms, the contact of the bag contents (especially zeolite powders) with the atmosphere was reduced to limit a potential loss of adsorption performance.

**Figure 9:**
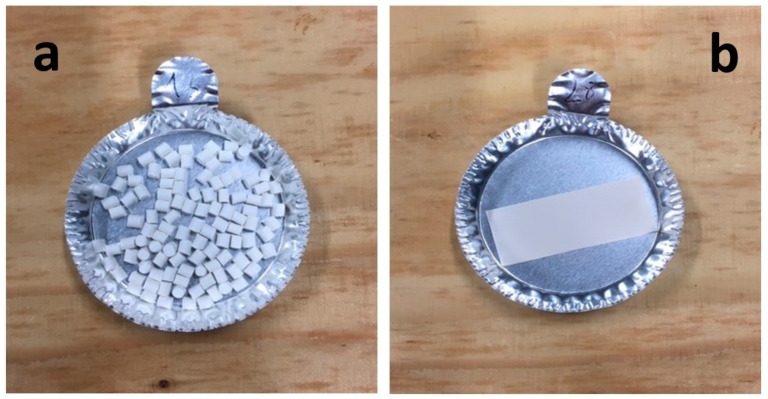
Sample layout for (**a**) granulates and (**b**) molded part samples.

Manufacturing of composite materials into the granulate form was conducted using a mixing and blending process, see Figure 9a. Granulates were then used for the manufacturing of the composite materials into form of molded parts using the injection process with a Billion Dixit 2 Proxima H120-500 injection molding machine (mold cavity, length = 208 mm and thickness = 0.8 mm), see Figure 9b. After manufacturing, the samples were characterized to determine their zeolite loading, the distribution of zeolite crystals in their composite bodies, and their water adsorption capacities. The obtained composite material samples were denoted as follows: cA-Cg or cA-Cmp with c the major compensating cation of the contained zeolite, C = composite, g = granulate form and mp = molded part form. For example, MgA means zeolite A exchanged with MgCl_2_ aqueous solution, while MgA-Cg means composite material in granulate form containing zeolite A exchanged with MgCl_2_ aqueous solution.

### 3.5. Characterization Techniques

#### 3.5.1. X-ray Fluorescence (XRF)

Chemical analyses were performed using an X-ray Fluorescence (XRF) spectrometer (PANalytical Zetium (4 kW) on samples previously pressed into 13 mm diameter pellets for 10 min at a pressure of 5 tons.

#### 3.5.2. Inductive Coupled Plasma Optical Emission Spectroscopy (ICP-OES)

The samples underwent acid digestion at room temperature for 24 h (0.05 g of sample + 3 mL of 48.9% Fluoridic Acid (HF)). The solution thus obtained was diluted to 30 mL and was then filtered at 0.45 μm before analysis using Thermo ICAP 6300 DUO equipment.

#### 3.5.3. X-ray Diffraction (XRD)

The X-ray diffraction patterns were recorded on a PANalytical MPD X’Pert Pro diffractometer operating with Cu Kα radiation (Kα = 0.15418 nm) equipped with an X’Celerator real-time multiple strip detector (active length = 2.12° 2θ). The XRD powder patterns were collected at 22 °C in the 3° < 2θ < 50° 2θ range, with a step of 0.017° in 2θ and a time of 220 s per step.

#### 3.5.4. Scanning Electron Microscopy (SEM) and Energy Dispersive X-Rays Spectroscopy (EDX)

Scanning Electron Microscopy micrographs and Energy Dispersive X-Rays spectroscopy mappings were obtained on a Philips XL 30 FEG microscope. Before analysis, the samples were coated with a fine carbon layer using a BAL-TEC SCD004 sputter coating system in order to improve the electrical conductivity. Composite samples (granulates and molded parts) were immersed in a polymer resin before being analyzed. After hardening, the polymer resin was polished until cutting the samples in one plan to obtain a representation of the zeolite crystal distribution.

#### 3.5.5. Solid-State Nuclear Magnetic Resonance (Solid-State NMR)

^29^Si solid-state Magic Angle Spinning (MAS) NMR spectra with ^1^H decoupling were recorded on a Bruker AVANCE II 300WB spectrometer (B0 = 7.1 T) operating at 59.59 MHz with a 2.6 µs pulse duration corresponding to a flip angle of π/6 and 80 s of recycling delay. Samples were packed in a 7 mm cylindrical zirconia rotor and spun at a spinning frequency of 4 kHz. ^29^Si chemical shifts were referenced to tetramethylsilane (TMS). ^27^Al MAS NMR spectra were recorded on a Bruker AVANCE II 400WB spectrometer (B0 = 9.4 T) operating at 104.2 MHz using a 2.5 mm cylindrical zirconia rotor and spun at a spinning frequency of 12 kHz. ^27^Al chemical shifts were given relative to an aqueous solution of aluminum nitrate (Al(NO_3_)_3_). Typical acquisition parameters included a pulse duration of 0.3 μs corresponding to a flip angle of π/12 and 1 s recycle delay. Decompositions of the NMR spectra to extract the proportion of the corresponding species were performed with the DMfit software [77].

#### 3.5.6. Determination of the Zeolite Loading into Composite Materials Formulations

The amount of raw and exchanged zeolite samples contained in the composite materials were determined using a Nabertherm Controller B170 oven. Samples were taken from the sealed aluminum bags in which the composite materials were directly stored and protected after being manufactured. To determine the zeolite loading, a calcination program was conducted, see Table 8. Heating the composite sample caused the decomposition of the organic matter, whereas the inorganic part was stable until 700 °C. By weight difference, the inorganic content corresponding to zeolite in the formulation could be obtained.

#### 3.5.7. N_2_ Adsorption-Desorption Measurements

The textural characteristics of raw and exchanged powder zeolite samples were determined from the N_2_ adsorption–desorption isotherms performed at −196 °C using a Micromeritics ASAP2420 instrument. Prior to the sorption measurement, the samples (50–100 mg) were outgassed under a vacuum at 90 °C for 1 h and 300 °C for 15 h to remove the physisorbed water (zeolite powder). The Brunauer–Emmett–Teller specific surface area (S_BET_) was calculated by using the BET method, while the t-plot method was used to determine the sample microporous volume (Vm).

#### 3.5.8. Water Adsorption Measurement

– Water adsorption isotherms of raw and exchanged zeolite powder samples were performed at 25 °C using a Micromeritics ASAP 2020 instrument. Prior to the water adsorption measurements, water (analyte) was flash frozen with liquid nitrogen and was then evacuated under a dynamic vacuum at least 5 times to remove any gases in the water reservoir. The samples (50–100 mg) were outgassed under a vacuum at 90 °C for 1 h and at 300 °C for 24 h to remove the physisorbed water. The water adsorption capacity of the samples was determined from the water adsorption isotherms at p/p^0^ = 0.2.
– The water adsorption kinetics of the raw material, the exchanged zeolites, and the composite materials containing the raw and exchanged zeolites (NaA and MgA) were performed by following the weight variation along the time at 30 °C at a relative humidity of 80% using a Memmert HCP 108 humidity chamber. Samples were taken from the sealed bags in which they were stored after being manufactured to avoid moisture intake and were directly used for measurements.

## 4. Conclusions

Before the elaboration of the LTA-type zeolite-polymer composite, the NaA zeolite in its powder form exchanged at large scale with magnesium cations was fully characterized.

X-ray Fluorescence (XRF) and Energy Dispersive X-Ray spectroscopy (EDX) analyses showed that a significant amount of the introduced Mg^2+^ cations were homogeneously distributed into the zeolite framework, a sign of a successful cationic exchange. Scanning electron microscopy photographs highlight the cubic morphology of LTA crystals, while XRD and NMR analysis confirmed that the exchanged zeolite does not suffer from significant structural changes.

The replacement of sodium cations by a smaller divalent cation such as magnesium leads to the increase of the available microporous volume, which, in addition to less congested pore opening, increases the accessibility to the micropores leading to an improved storage of the host molecules, as is the case in water. In addition, divalent cations lead to a higher degree of ordered water molecules around them, and a better spatial organization also contributes to improving water adsorption. According to the results of the water adsorption isotherms, an increase of the water adsorption capacities of 32% was observed for magnesium exchanged sample (MgA zeolite) in comparison to the parent material NaA zeolite indicating, that a magnesium cation offers higher performance.

The MgA zeolite was mixed with polymers to manufacture composite materials in granulate and molded part forms using extrusion and injection processes, respectively. The determination of the zeolite loading in the respective composite formulations showed consistent results with the introduced amounts of components into the formulations, which points toward homogeneous and viable shaping processes. This is confirmed through SEM analysis, which showed that a homogeneous distribution of zeolite crystals into the polymers matrix for both granulates and molded part forms accompanied the preferential orientation of polymer fibers and zeolite crystals.

Water adsorption was observed for each composite, regardless of the shape and the nature of the charge compensating cations contained in the zeolite. The water adsorption capacities of the zeolite containing magnesium are higher than those of the sodium form by about +22%, +27% and +18% for the powder, granulate and molded part forms, respectively. This work confirmed that the developed composite materials with zeolites as fillers preserved the adsorption properties of the zeolite crystals. These exchanged zeolites are promising for uses in water decontamination applications because of either an increased protection against water or a smaller amount of adsorption required to trap the same amount of water compared to their sodium counterparts.

In addition, the shaping of the zeolites into different forms changes the water adsorption kinetics of composite materials, which could be used to tune these properties depending on the targeted adsorption applications. 

## Figures and Tables

**Table 1:** Chemical composition of LTA-type zeolite and its associated magnesium form determined by XRF (Si/Al, Na/Al, Mg/Al, and K/Al Molar ratio).

Samples	Molar Ratio ^1,2^			Number of Positive Charges per Al Atom
	Si/Al	Na/Al	Mg/Al	(Na + K + 2 Mg)/Al
NaA	1	1.07	0	1.09
MgA	1	0.45	0.29	1.05

^1^ Experimental error 3%. ^2^ Average of 3 measurements.

**Table 2:** Rate of exchange, BET surface area (SBET), microporous volume (Vm), and water adsorption capacity for the raw and exchanged LTA-type zeolite.

Samples	Rate of Exchange ^a^ (%)	S_BET_^b^ (m^2^·g^−1^)	V_m_ ^c^ (cm^3^·g^−1^)	Water Adsorption Capacity ^d^ (mmol·g^−1^)	Water Adsorption Capacity ^e^ (Wt.%)
NaA	0	x	x	12.1 (±0.4) ^f^	21.8 (±0.7) ^f^
MgA	58	693	0.26	16.0 (±0.5) ^f^	28.8 (±0.9) ^f^

x: Not porous to nitrogen probe molecule. ^a^ Rate of exchanged sodium cations. Value determined by the XRF measurement. ^b^ Value determined by the BET method (average of 3 measurements). ^c^ Value determined by the t-plot method (average of 3 measurements). ^d^ Value determined from water adsorption isotherm at 25 °C (taken at p/p^0^ = 0.2) (average of 2 measurements). ^e^ Value obtained by multiplying the amount of adsorbed water in mmol·g^−1^ by the molecular weight of water MW = 18.02 g.mol^−1^ (average of 2 measurements). ^f^ Standard deviation.

**Table 3:** Composite material samples containing NaA and MgA zeolite samples in both granulates and molded part forms.

Used Zeolite	Granulate Form	Molded Part Form
NaA	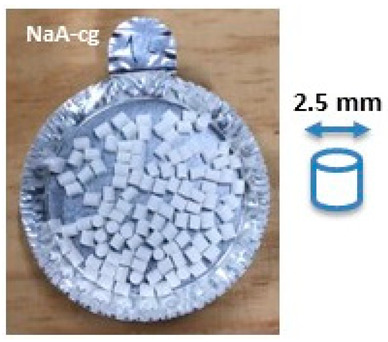	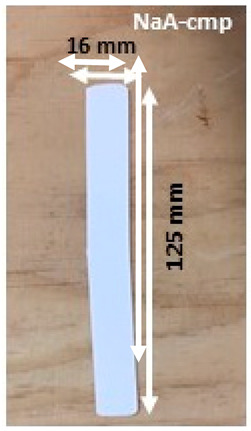
MgA	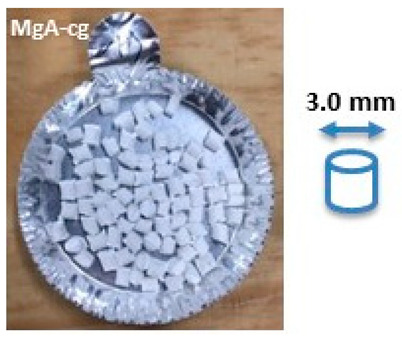	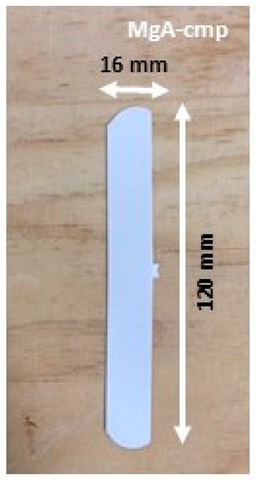

**Table 4:** EDX mapping of carbon, silicon, aluminum, sodium, and magnesium of the NaA-cg and MgA-cg samples.

	NaA-cg	MgA-cg
Crystals	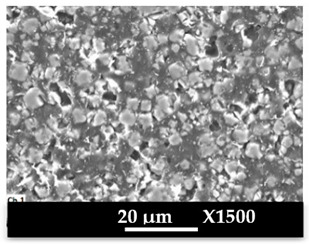	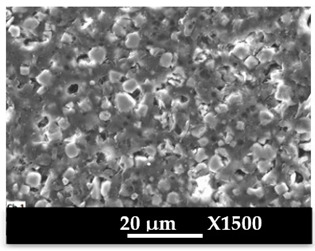
Carbon	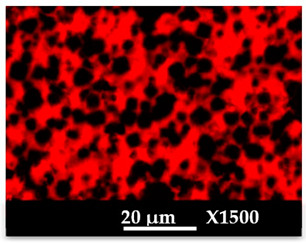	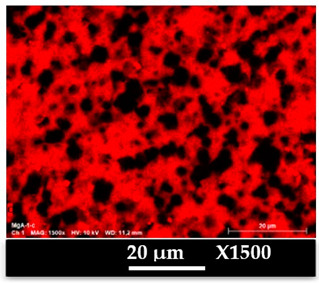
Silicon	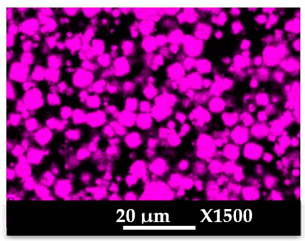	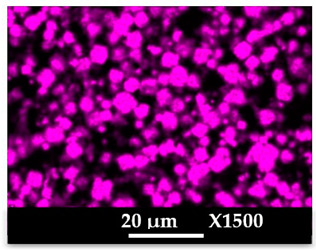
Aluminum	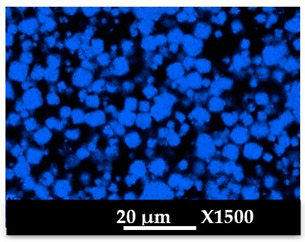	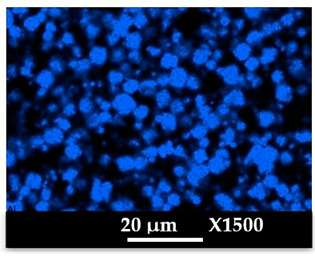
Sodium	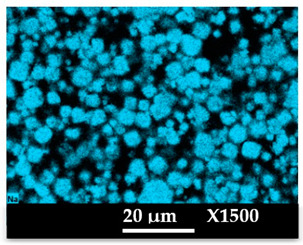	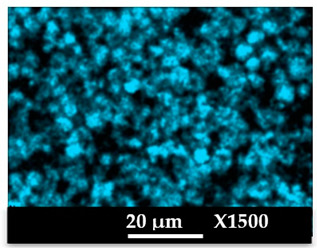
Magnesium		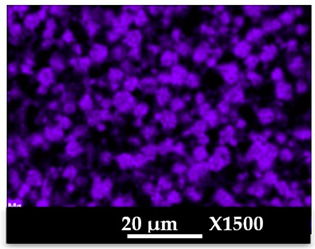

**Table 5:** EDX mapping of carbon, silicon, aluminum, sodium, and magnesium of the NaA-cmp and MgA-cmp (side view) samples.

	NaA-cmp	MgA-cmp
Crystals	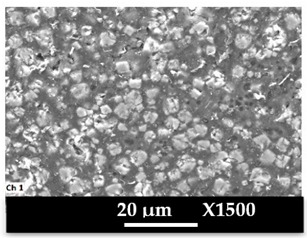	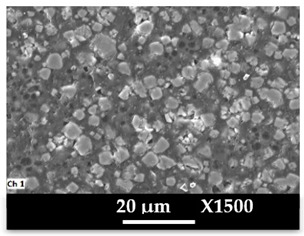
Carbon	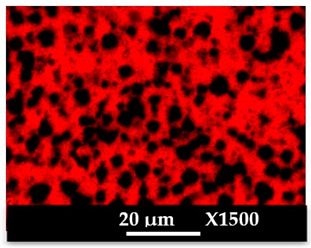	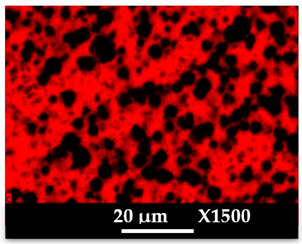
Silicon	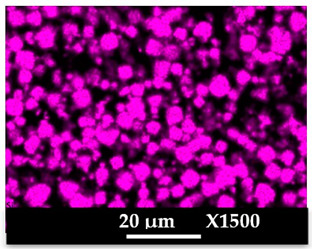	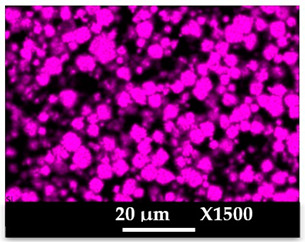
Aluminum	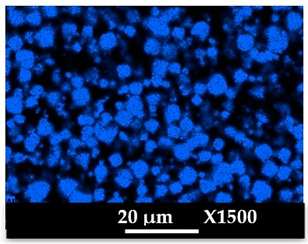	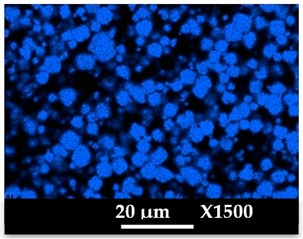
Sodium	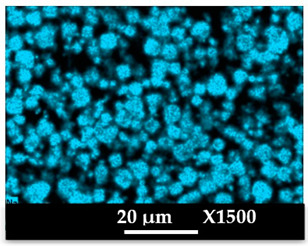	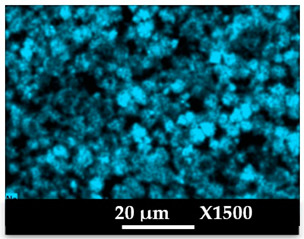
Magnesium		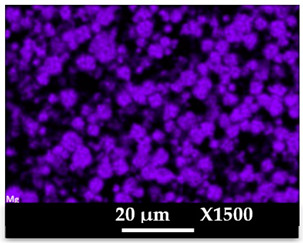

**Table 6:** Water adsorption capacity of the NaA and MgA zeolite samples and their associated composite forms at 30 °C.

	Water Adsorption Capacity (Wt.%)			
	Powder ^1^	Powder ^2,3^	Granulate ^2,3,4^	Molded Part ^2,3,4^
NaA	21.8 (±0.7) ^5^	23.5 (±0.2) ^5^	24.0 (±0.4) ^5^	23.3 (±0.3) ^5^
MgA	28.8 (±0.04) ^5^	28.6 (±0.2) ^5^	30.4 (±1.0) ^5^	27.5 (±0.1) ^5^

^1^ Results from Table 2. Measurements performed using ASAP2020 equipment (isotherms of water adsorption at 25 °C). ^2^ Measurements performed in the humidity chamber (80% RH—30 °C). Prior to the measurements, a degassing step based on the conditions reported in Table 7 was performed. ^3^ Average of three measurements. ^4^ The water adsorption capacity is calculated for 100% of activated zeolite by taking into account the zeolite loading. ^5^ Standard deviation.

**Table 7:** Oven temperature program for the dehydration of zeolite powder samples.

	Temperature (°C)	Time (h)	Observations
Heating Phase	20 ➔ 500	8	Temperature rate ≈ 1 °C/min
	500	6	Holding time
Cooling Phase	500 ➔ 200		Cooling until 200 °C.

**Table 8:** Oven temperature program for the calcination of composite materials.

	Temperature (°C)	Time (h)	Observations
	30	20	Holding time
Heating Phase	30 ➔ 650	620	Temperature rate ≈ 1 °C/min
	650 ➔ 120		Holding time
Cooling Phase	650 ➔ 300		Until 300 °C and then transferred into a desiccator containing phosphorus pentoxyde (P_2_O_5_) to avoid the trapping of water by the zeolite. After 10 min, the sample is weighed.

## Data Availability

Data are available from the authors upon reasonable request.
